# The Formation of Protein Corona by Nanoplastics and Horseradish Peroxidase

**DOI:** 10.3390/nano12244467

**Published:** 2022-12-16

**Authors:** Jing Zhou, Yanni Yu, Yaning Luan, Wei Dai

**Affiliations:** The Key Laboratory for Silviculture and Conservation of Ministry of Education, College of Forestry, Beijing Forestry University, Beijing 100083, China

**Keywords:** nanoparticle, polystyrene, enzyme, protein corona, characterization

## Abstract

In theory, nanoplastics (NPs) can adsorb biological macromolecules, such as proteins, in the surrounding environment to form protein corona (PC). In this study, we focus on amino polystyrene (PS) NPs and horseradish peroxidase (HRP) to explore the dynamic process of the formation of PS-HRP PC and their influence on PS and HRP. This work used atomic force microscopy, laser particle size and Zeta potential analyzer, and UV-vis spectrophotometer. According to the adsorption behavior of HRP to NPs, the surface morphology characteristics of NPs can be observed to change at 60 min. Meanwhile, the increase in size and hydrodynamic diameter, the decrease in Zeta potential, surface roughness and HRP activity, and the change in HRP structure attest to the PC formation. The thickness of the PC was approximately 30 nm and there are differences in the dynamic and static variations in the size of the PC. The PC formation process progresses gradually from 0 min to 240 min. Overall, the formation of PS-HRP PC is identified, and the changes in its properties are confirmed from the perspective of nanoplastics and peroxidase, which help study the effects of nanoplastics on the environment and creatures.

## 1. Introduction

Nanoplastics (NPs), a major global environmental problem [1], are more damaging [2] due to their size and features and pose a potential threat to biosecurity [3], human health [4], and so on. Nanoparticles, including NPs, have a negative impact on plants and animals through the accumulation and transmission of food chains [5]. Because of their nanoscale, large specific surface area, and strong adsorption, nanoparticles may interact with biomolecules such as proteins, nucleic acids, lipids, and other biological metabolites in the biological environment [6], forming a corona on their surface called “protein corona” [7]. On the one hand, the formation of the protein corona (PC) may alter the size, surface charge, aggregation state, and other structural properties of nanoparticles, directly or indirectly leading to biological properties that differ from the original ones [8,9], which is mainly because the adsorbed protein further modifies nanoparticles’ surface [10], so the nanoparticles’ interaction with protein is controlled [11]. On the other hand, the formation of the PC may alter protein conformation, exposing new novel epitopes and producing avidity effects arising from the close spatial repetition of the same protein [12]. The PC may also disrupt protein structures [13], causing a perturbed function triggered by structural effects or local high concentration, which may further affect its agglomeration, absorption, and toxicity [14]. For instance, the formation of the PC mitigates the toxic effects of different surface-modified PS NPs towards marine microalgae *Chlorella* sp. [15].

Polystyrene (PS) NPs have a unique adsorption capability, high surface area to volume ratio, high surface curvature, and high mobile properties. Thus, they are widely used to produce a variety of consumer products in life, such as food packaging, nanocomposites, nanomedicine, and cosmetics [16]. Additionally, it can be exposed to the environment for a long time, so it deserves widespread attention. Generally, positively charged NPs (PS-NH_2_) induced stronger toxicity than bare NPs and negatively charged NPs (carboxyl-modified PS, sulfonic acid-modified PS and epoxide-modified PS) [17]. Horseradish peroxidase (HRP) is a common and extensively studied oxidoreductase, a glycoprotein containing heme as a prosthetic group [18], which widely exists in plants and soils, catalyzing both oxidation and the reduction of a variety of compounds and peroxides, respectively, and playing a potential role in the soil detoxification [19]. The remarkable catalytic activity, availability, and high stability of HRP in an aqueous solvent make it a model enzyme for exploring the underlying structure-function relationships [20]. The chemical essence of HRP is protein, and its conformational characteristics play a vital role in physiological functions, such as the immunomodulation of living organisms, and is the direct influencing factor for maintaining the normal activities of life. The adsorption of proteins on the surface of nanoparticles was recognized decades ago [21] and has shown great potential for applications in biomedical and life sciences fields. However, the cognition of the PC formation is still currently limited, and the relevant scientific questions are waiting to be solved.

Plants are likely to absorb microplastics and nanoplastics from the environment, affecting growth [22]. Once plastics are taken up and transported by plants, they may interact with enzymes in vivo to form the PC, resulting in affecting its activity and distribution and the biotoxicity level of plastics through the formation of new conformations and binging points. Since peroxidase is widely distributed in the environment and performs important functions, it is of great importance to clarify its interactions with the surrounding environment and organisms. Hence, in this study, we focus on HRP and PS NPs to explore the formation and characteristics of the PC by nanoplastics and peroxidase in vitro, which will provide some favorable supplements for studying the toxicity of nanoplastics and the formation of protein corona in plants, soil, and other environments. It may better present the interaction process of a single type of plastic and enzyme in a simple environment. The formation and characteristics of the certain PS-HRP PC are described through microscopic imaging, absorption features detection, and so on.

## 2. Materials and Methods

### 2.1. Materials

PS NPs have a size of 200 nm and are modified with amino (-NH_2_) on the surface. They are positively electrical, hydrophilic, and can be evenly dispersed in deionized water. The optimal catalytic pH of HRP (RZ > 1.5, activity > 100 IU/mg) is 7.0, and the isoelectric point (pI) is 7.2. All other chemicals used are of analytical grade.

### 2.2. Synthesis of PS-HRP PC

This experiment sets up an experimental group and a control group. Mix 1.0 mg/mL PS suspension with an equal volume of 0.5 mg/mL HRP solution in the experimental group, while set PS suspension and HRP solution mixed with ultrapure water respectively as the control group. Then place the mixed solutions in a water bath thermostatic shaker (QIANG LE, Jiangsu, China, SHZ-88A) and incubate them under the condition of 25 °C and 100 r/min for 10 min, 30 min, 60 min, and 240 min, respectively. The different incubation times are set based on that the commonly accepted time scale to obtain a stable composition of the hard corona is 1 h of incubation [23]. Then place the suspension in a refrigerated ultracentrifuge (RUI JIANG, Jiangsu, China, RJ-TGL-1850R) and centrifuge at 15,300× *g* centrifugal force and 4 °C for 15 min. Collect the supernatant, add appropriate ultrapure water to the pellet, and centrifuge again. This step is repeated three times to remove the free HRP that is not bound to NPs in the PC suspension as much as possible. Collect the supernatant after each centrifugation in the same container and re-disperse the purified pellet into ultrapure water. Centrifugation is the most common and established method to isolate nanocarriers with their PC, which are strongly bound proteins from the surrounding medium [24], and are considered “hard corona”.

### 2.3. Determination of Adsorption of PS to HRP

Mix and oscillate purified PC supernatant and Coomassie Brilliant Blue G-250 solution. Use an ultraviolet spectrophotometer (AOE, Shanghai, China, UV-1800) to determine the absorbance at 595 nm 2 min later. The concentration of HRP in the PC supernatant is found from the corresponding standard curve according to the A_595_, which is plotted with the solution concentration (mg/mL) of standard bovine serum albumin (BSA) as the abscissa and the absorbance value A_595_ as the ordinate. The adsorption rate of PS to HRP is calculated using the following formula:

(1) $$Adsorption\;rate\;(\%)\;=[C_{HRP}\;-\;T_{HRP})/C_{HRP}]\;\times \;100$$

where $C_{HRP}$ and $T_{HRP}$ is the concentration of HRP in the control and experimental group.

### 2.4. Atomic Force Microscope (AFM) Images Analysis

Take a drop of PS solution and the PC suspension with a suitable concentration, spread it on the mica sheet, and let it dry naturally. Samples are treated with ultrasonication for 5 min before the sectioning production. Then scan samples in tapping mode by atomic force microscopy (Bruker, Karlsruhe, Germany, Multimode 8) and process and analyze the acquired AFM images with NanoScope Analysis 1.5 to observe the morphological characteristics of the PC. Thirty particles are randomly selected each time in AFM images to measure their size and roughness. The diameter distribution is statistically calculated based on the measurement results of size. The mean and standard error for different treatments is calculated, and the analysis of ANOVA (LSD test method, *p* < 0.05) is performed.

### 2.5. Zeta Potential, Hydrodynamic Diameter, and Polydispersity Index Measurement

Determine Zeta potential, hydrodynamic diameter, and polydispersity index of PS NPs and the PC with a disposable sizing cuvette on a laser particle size and a Zeta potential analyzer (Malvern, Worcestershire, U.K., Zetasizer Nano ZS90) at 25 °C. Measure Zeta potential by the method of electrophoretic light scattering, while hydrodynamic diameter and polydispersity by the dynamic light scattering method. Samples are treated with ultrasonication for 5 min before the assay. The measurement is performed at least three times.

### 2.6. HRP Activity Assay

Take three centrifuge tubes for each assay. Add the deactivated solution first boiled in a boiling water bath for 5 min for inactivation treatment to one tube ($A_{0}$) as the blank control and the solution without other treatment to two tubes ($A_{1}\;$and $A_{2}$) as experimental ones. Then add 1× PBS buffer, 0.1 mol/L hydrogen peroxide (H_2_O_2_) solution, and 0.05 mol/L guaiacol solution to each tube. Incubate all reaction solutions at 25 °C for 3 min and pour them into the quartz cuvettes quickly. Determine the absorbance at 436 nm using an ultraviolet spectrophotometer (AOE, Shanghai, China, UV-1800). Read the absorbance value A_436_ every minute, and a total of 4 min was measured. The one activity unit (u) of HRP is counted by the amount of enzyme that will reduce the absorbance value A_436_ by 0.01. The relative activity of the HRP that adsorbed to the surface of PS NPs is calculated using the following formulas:

(2) $$∆A_{436}=A_{0}\;-\;(A_{1}+A_{2})/2$$

(3) $$HRP\;activity\;(u/\min)\;=\;∆A_{436}/(0.01\times t)$$

(4) $$Relative\;activity\;=\;(T_{a}/C_{a})\times 100$$

where $A_{0}$, $A_{1}$, and $A_{2}\;$is absorbance value $A_{436}$ of the corresponding reaction solution. $C_{a}$ is the activity of HRP before incubation and $T_{a}$ is that after incubation.

### 2.7. UV-Vis Absorption Spectra

Test and record UV-vis absorption spectra of HRP, PS, and the PC were recorded from 190 nm to 800 nm with supernatant in a quartz cuvette from 190 nm to 800 nm on a UV-vis spectrophotometer (Shimadzu, Kyoto City, Japan, UV-2600) tested with supernatant in a quartz cuvette.

### 2.8. Statistical Analysis

All measurements were conducted at least in triplicate. The statistical analysis and graphical outputs were conducted using Microsoft Excel 2019 and Origin Pro 2018. One-way analysis of variance (ANOVA) was performed among all the treatments using Duncan’s multiple comparisons with a significance level of *p* < 0.05.

## 3. Results and Discussion

### 3.1. Adsorption of HRP to PS NPs

**Figure 1:**
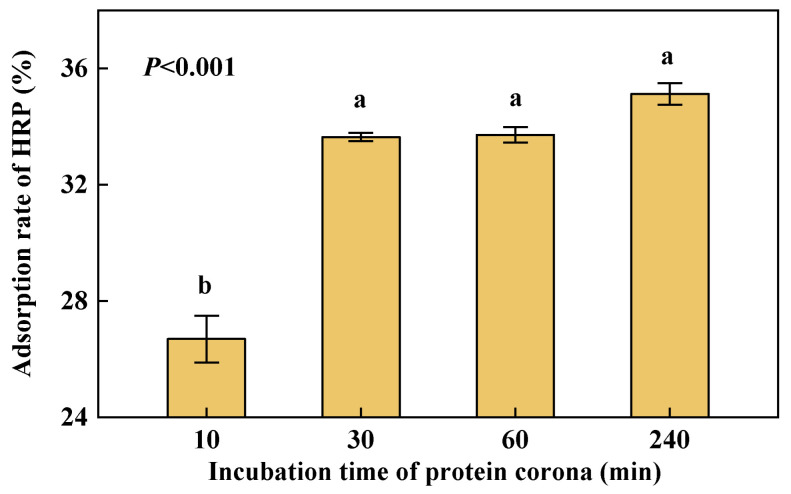
Adsorption rate changes of PS NPs to HRP over time. Dates are means *±* SE (*n* = 3). Different letters indicate a significant difference at *p* < 0.05.

The results showed that the concentration of HRP in the supernatant after incubation and purification decreased significantly compared with the control one. According to Figure 1, NPs absorbed about 26.9% HRP at least, indicating that HRP adsorbed on PS NPs and probably formed the PC. In addition, during 0 min~10 min and 10 min~30 min, PS NPs rapidly absorbed free HRP from the solution due to their large surface and interaction forces between substances. After 30 min, the adsorption rate of HRP by NPs tended to flatten. As the adsorption rate changes over time, the interaction process between NPs and HRP gradually reach a relatively balanced and stable state.

### 3.2. Morphology of PS-HRP PC

**Figure 2:**
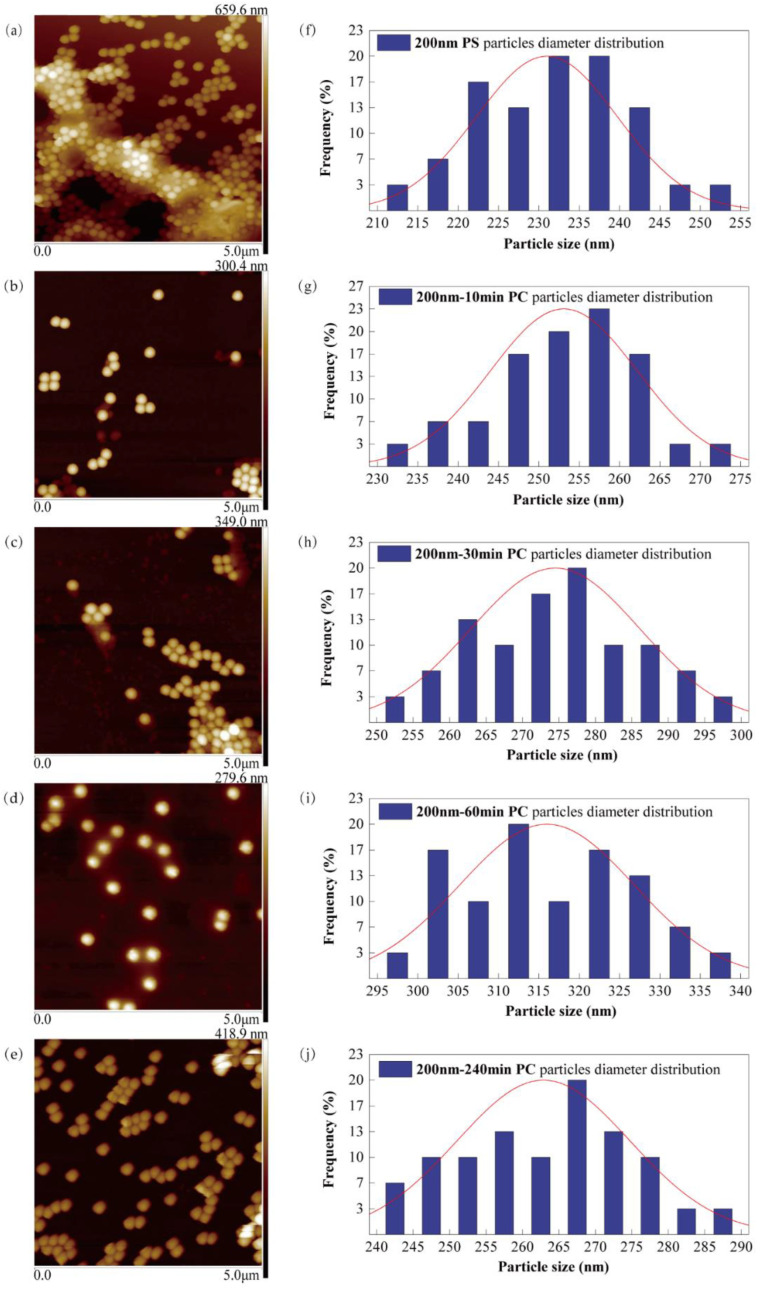
AFM images and diameter distribution of PS NPs (**a**,**f**) and PS-HRP PC incubated for 10 min (**b**,**g**), 30 min (**c**,**h**), 60 min (**d**,**i**), and 240 min (**e**,**j**).

To explicit the formation of PS-HRP PC and further investigate the appearance differences between NPs and the PC, AFM images were processed to observe the surface characteristics (Figure 2a–f). AFM images showed that the surface morphology of PS NPs changed after being wrapped by HRP compared with the pristine NPs. In particular, at 60 min, the NPs’ surface was observed to be attached by a layer of HRP, and the edge was blurred. However, the shape of NPs remained approximately spherical, which was not affected by HRP. The description above illustrates that HRP was adsorbed and covered on the surface of NPs, indicating the formation and existence of the PC. In addition, the PC caused NPs to change the surface morphological characteristics but maintain their pristine shape. The particle diameter distribution chart (Figure 2f–j) showed that the diameter distribution of NPs and the PC was normally distributed. This indicates that the diameter measurement results are representative and reliable.

### 3.3. Changes in Size and Hydrodynamic Diameter of NPs

**Figure 3:**
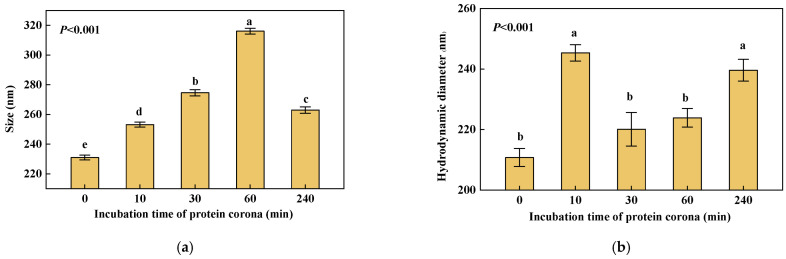
Size (**a**) and hydrodynamic diameter (**b**) changes of PS NPs and the PC over time. Dates are means *±* SE (*n* = 3~30). Different letters indicate a significant difference at *p* < 0.05.

According to the measurement of AFM images, the 200 nm PS NPs have a size of 231.0 ± 1.6 nm. The size of NPs increased by 22.1 nm~85.0 nm once they interacted with HRP (Figure 3a), indicating the formation of the PC. The size was constantly getting larger during 0 min~60 min and reached a max size of 316.0 ± 2.0 nm. However, during 60 min~240 min, the size became smaller. What is above was consistent with that the most obvious morphological changes can be observed at 60 min (Figure 2d). Although HRP constantly adsorbed on NPs over time, the size did not increase all the time. This indicates the PC formed on the surface of NPs might undergo dynamic progress of change. The PC may go through a process of the monolayer to the multilayer and then to the monolayer during incubation. Finally, a near-monolayer of biomolecules binds tightly, but not completely irreversibly, to the PS NPs’ surface [25].

The results showed that the hydrodynamic diameter of the pristine 200 nm PS NPs is 210.8 ± 3.0 nm. After interacting with HRP, the hydrodynamic diameter increased by approximately 10 nm~35 nm (Figure 3b). The formation of PS-HRP PC significantly increased the hydrodynamic diameter with the same result as the size. The hydrodynamic diameter of NPs increased from 0 min to 10 min and then decreased from 10 min to 30 min. During 30 min~240 min, the hydrodynamic diameter increased again. Once PS NPs and HRP contacted during the incubation, they were rapidly attracted to each other due to the interaction force, resulting in a large amount of free HRP gathered around NPs to make a Brownian motion together. The above may account for the large hydrodynamic diameter at 10 min. Then with more and more HRP adsorbed on NPs, the PC formed on the surface, which may inhibit particle-to-particle interactions and reduce the influence of free HRP on the hydrodynamic diameter. After 30 min, the hydrodynamic diameter increased slowly to about 240 nm. DLS measurement results varied because the particles assayed and wrapped by the corona are different in composition and surface chemistry than those originally synthesized, which have a surface [26]. At 240 min, the increase in the hydrodynamic diameter of NPs was consistent with the increase in size, both by about 30 nm, which indicated that the thickness of PS-HRP PC was approximately 30 nm.

In general, average DLS values for characterizing mono size dispersed polystyrene nanoparticles are slightly higher than that of AFM values [27]. However, the results of this study are contrary to what was reported. The hydrodynamic diameter of PS NPs is lower than that of dried ones measured by AFM lacking hydration. The reasons should be further explored through experiments to determine whether the existence of this phenomenon is reasonable and the reasons for it, such as materials, functional groups, etc.

### 3.4. Changes in Zeta Potential of NPs

**Figure 4:**
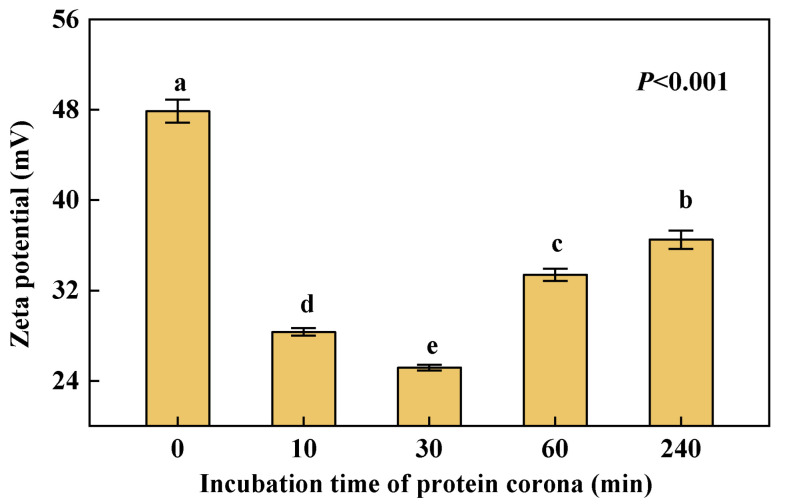
Zeta potential changes of PS NPs and the PC over time. Dates are means *±* SE (*n* = 3). Different letters indicate a significant difference at *p* < 0.05.

According to Figure 4, PS NPs are all positively charged in the aqueous solution regardless of whether or not they interact with HRP. The Zeta potential of the pristine NPs in the control group was 47.9 ± 1.0 mV, and the absolute value decreased significantly after contact with HRP. The formation of the PC reduced charges on NPs’ surface and decreased the stability of the dispersion system. In addition, Zeta potential showed a downward trend from 0 min to 30 min and an upward trend from 30 min to 240 min. At 30 min, Zeta potential reached a minimum value of 25.2 ± 0.3 mV, and the solution system was in the most unstable state. The stability of PS NPs was destroyed by HRP during the initial formation of the PC. Then, through the densification process of the PC, the stability of the system gradually recovered and stabilized again. Studies have shown that the colloid was stable at a point where enough proteins were present in the solution to cover all nanoparticles’ surfaces in a monolayer fashion [28]. The PC formation can enhance particle stability by forming a layer between the nanoparticles and thus the solvent interfering with interparticle interactions and reducing or inhibiting agglomeration [29].

### 3.5. Changes in Surface Roughness of NPs

**Figure 5:**
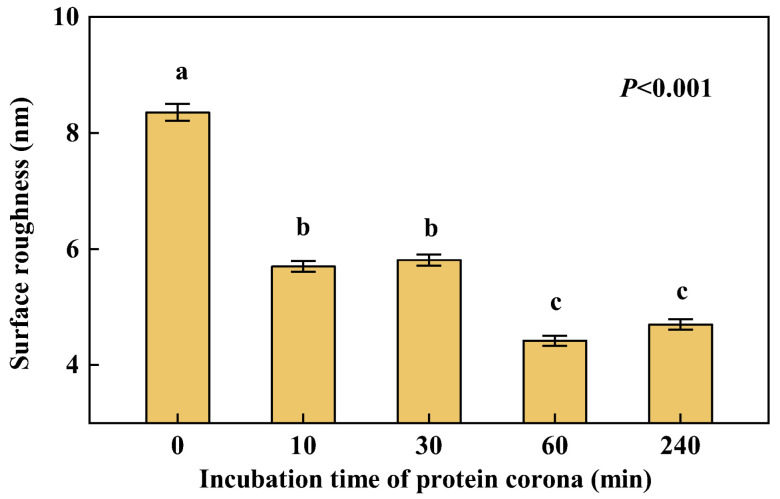
Surface roughness changes of PS NPs and the PC over time. Dates are means ± SE (*n* = 30). Different letters indicate a significant difference at *p* < 0.05.

The results show that the surface roughness of PS NPs has reduced from 8.35 nm ± 0.15 nm to about 4 nm after the formation of the PC (Figure 5) and indicate that the formation of PS-HRP PC changed the surface of NPs from relatively rough to smooth. The functional amino (-NH_2_) group modified on the surface of NPs may increase the surface roughness. The surface of NPs was then wrapped by more and more HRP, as well as changes in HRP conformation and structure, leading to a decrease in surface roughness. In addition, the surface roughness of NPs showed a general downward trend over time. A noticeable decline in surface roughness was observed during 30 min~60 min. The above illustrated that the surface roughness changes significantly in the earlier and later stages of PS-HRP PC formation. It may be because the conformation and structure of the PC undergo qualitative changes in the later stage with more HRP attached to the NPs and the further interaction between them, such as the effect of the modified amino group.

### 3.6. Changes in Polydispersity Index (PDI) in of NPs

As shown in Table 1, the PDI of 200 nm PS NPs before and after the incubation was all about 0.05, and there was no significant difference. This indicated that the particle size distribution of PS NPs was uniform and almost unchanged throughout the process. The increase or decrease in the size of individual PS NPs under the same experimental treatment was uniform, indicating that HRP was evenly adsorbed on the surface of different NPs individuals. The formation of the PC was universal and consistent, and the determination and characterization of its properties had representativeness.

### 3.7. Changes in Activity of HRP

**Figure 6:**
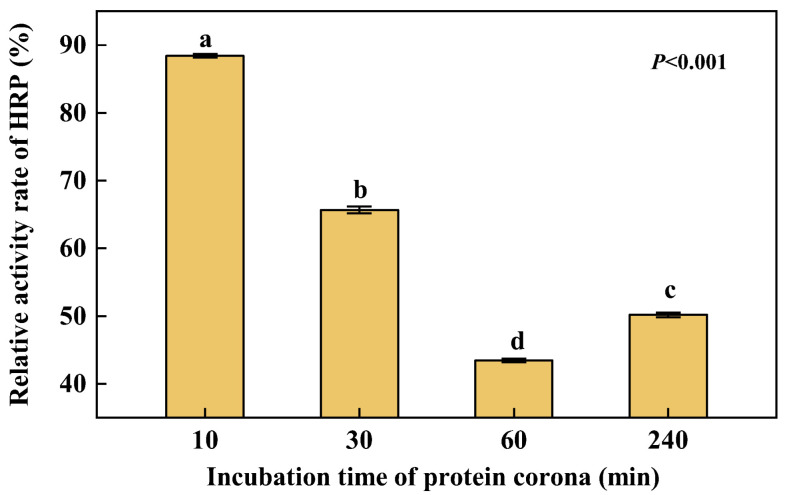
Relative activity changes of HRP with incubation time. Dates are means *±* SE (*n* = 3). Different letters indicate a significant difference at *p* < 0.05.

According to Figure 6, the interaction with PS NPs would lead to a decrease in HRP activity. From 0 min to 60 min, the relative activity of HRP gradually decreased and from 60 min to 240 min it recovered a little. At 60 min, HRP activity was reduced to a minimum, with only 43% activity of pristine ones. The interaction with the surface of particles leading to a decrease in enzyme activity is consistent with the conclusion in the literature [30]. In this work, at least 40% of the free HRP activity was retained after it was adsorbed onto PS NPs. This is a higher level of enzyme activity than that reported in the literature [30], where lysozyme adsorbed onto silica nanoparticles was found to retain at least 20% of its native activity in all systems studied. A decrease in enzyme activity may be due to the partial loss of its biochemical activity result of modification of its secondary structure, and the change of the enzyme’s active site may be one of the main reasons for the activity decrease [31].

### 3.8. Changes in Structure of HRP

**Figure 7:**
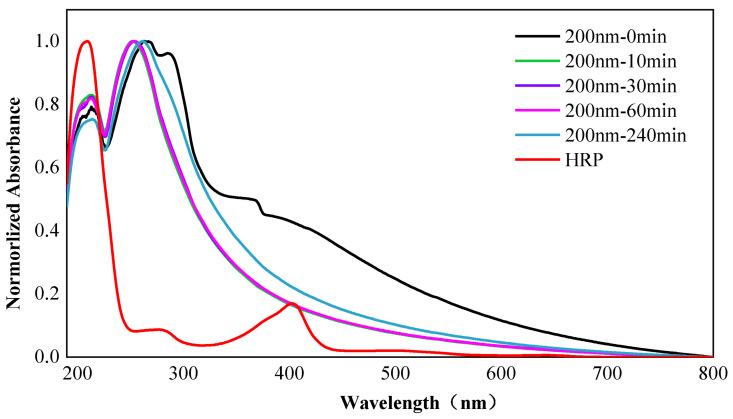
UV-vis adsorption spectra of PS NPs, HRP and PS-HRP PC over time.

The UV-vis spectrum is an effective tool for observing changes in the structure of HRP. In the study, the UV-vis absorption spectra are plotted based on the smoothed and normalized data. According to Figure 7, the UV-vis absorption spectra of PS-HRP PC with an incubation time of 10 min, 30 min, and 60 min are coincident, meaning that the structure essentially remains unchanged over time. When NPs are incubated with HRP for 10 min, the absorption peak of the UV-vis absorption spectrum undergoes an increase in intensity at a wavelength of 213 nm and a blue shift at 263 nm due to the formation of the PC. Then at 240 min, the absorption peak underwent a decrease in intensity at 213 nm and was weaker than that of pristine NPs. Meanwhile, a redshifted shift at 263 nm occurred and was located on the shorter wavelength side of pristine NPs. The redshift means the formation of a dense PC on the surface of NPs, which was consistent with the adsorption of proteins on the surface of NPs. Overall, the formation of PC has an effect on the structure of itself and HRP. Though the more significant conformational change is observed in the hard PC around smaller size nanoparticles compared to larger ones as the self-association forces hold the nanoplastics/protein complex [32]. NPs are prone to interact with proteins, and this interaction fundamentally changes the functionally crucial secondary structure of these biomolecules and thereby denatures them [33], which may lead to the misfolding of proteins and alterations in enzymes.

## 4. Conclusions

In conclusion, we focused on PS NPs and HRP to identify the formation of the certain PC, and confirm the changes in the properties, such as the surface morphology of NPs. The PC formation was clarified according to the adsorption behavior of PS NPs to HRP and the surface morphology characteristics of NPs showing the wrapped state, especially at 60 min. Meanwhile, the increase in size and hydrodynamic diameter, the decrease in Zeta potential, surface roughness and HRP activity, and the change in HRP structure attest to the formation and nature changes of the protein corona. The thickness of the formed PC was approximately 30 nm, according to the increase of 31.9 nm and 28.8 nm of NPs in size and hydrodynamic, respectively. The different characteristics of NPs and HRP over incubation time are indicated that the formation and nature changes of the PC occur gradually. Furthermore, there may exist a densification process from 60 min to 240 min to reach a relatively stable state in the protein canopy on the surface of NPs, supported by the increase in the number of adsorbed HRP, the decrease in the size, and the redshift of the UV-vis spectrum. The formation of the PC is confirmed, but it undergoes a dynamic evolution over time rather than formed at once. In addition, the affection of the PC on the structure of enzymes and the formation and properties of related PC in plants deserves to study more systematically and deeply in the future.

## Figures and Tables

**Table 1:** Polydispersity index (PDI) of PS NPs and the PC.

Incubation Time (min)	$PDI_{min}$	$PDI_{max}$	Means ± S.E.
0	0.003	0.077	0.046 ± 0.022
10	0.015	0.025	0.019 ± 0.003
30	0.011	0.068	0.046 ± 0.018
60	0.036	0.064	0.047 ± 0.009
240	0.043	0.078	0.059 ± 0.010

## Data Availability

Not applicable.
